# Ventricular trigeminy in a patient with serologically confirmed dengue haemorrhagic fever

**DOI:** 10.1186/1755-7682-7-28

**Published:** 2014-05-26

**Authors:** Anne Thushara Matthias, Jegarajah Indrakumar, Saman B Gunatilake

**Affiliations:** 1 University Medical Unit, Colombo South Teaching Hospital, Colombo, Sri Lanka; 2 Department of Medicine, University of Sri Jayewardenepura, Nugegoda, Sri Lanka

**Keywords:** Dengue fever, Ventricular trigeminy, Dengue myocarditis

## Abstract

**Background:**

Cardiac arrhythmias occur during the acute stage of Dengue Haemorrhagic Fever. Dengue myocarditis is the most likely cause of the arrhythmias.

**Case presentation:**

We report a 55-year-old patient with Dengue Haemorrhagic Fever presenting with transient ventricular trigeminy which has not been reported before.

**Conclusion:**

Among many other known cardiac arrhythmia seen in DHF, ventricular trigeminy is also a possibility. Clinicians should be aware of this cardiac rhythm abnormality that can occur in dengue patients.

## Background

Cardiac arrhythmias are uncommon in Dengue Haemorrhagic Fever (DHF). Cardiac rhythm disorders, such as atrioventricular blocks [1], ventricular ectopics [2] and atrial fibrillation [3] have been reported during episodes of DHF. Most of them are asymptomatic and have been self limiting with spontaneous resolution. The cause for these rhythm disorders are thought to be myocarditis. We report a case of ventricular trigeminy in a patient with DHF. Ventricular trigeminy complicating a serologically confirmed dengue fever is not reported before.

## Case presentation

**Figure 1:**
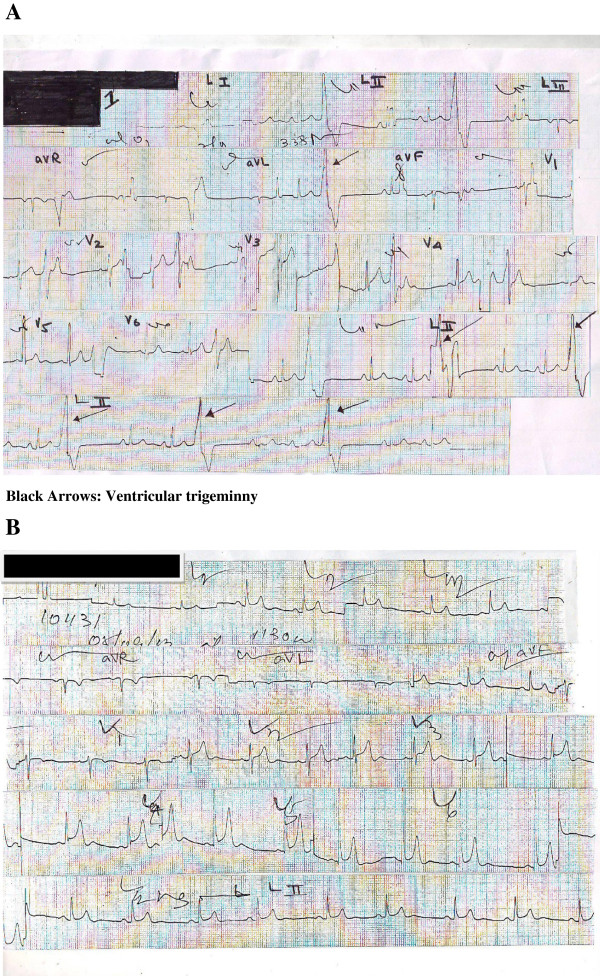
Electrocardiogram (A) Ventricular trigeminy (B) Sinus rhythm and normal ventricular complexes once ventricular trigeminy resolved.

A previously healthy 55-year-old man with no prior known cardiovascular disease was admitted with fever and arthralgia of five days. The patient had a temperature of 39°C and had an irregular pulse with a heart rate of 78 beats per minute. His blood pressure was 130/80. He had no other significant findings on examination. A 12-lead electrocardiogram showed ventricular trigeminy (Figure 1A).

The laboratory findings were: haemoglobin of 16.5 g/dL, a hematocrit of 48.1% and a white blood cell count of 3,100/mm^3.^ The platelet count was 95,000/mm^3^, reduced to 70,000/mm^3^ on the following day and 55,000/mm on the 5^th^ day. Other laboratory findings were potassium 4.6 mmol/L, sodium 135 mmol/L, magnesium 1.5 mg/dL, calcium9.6 mg/dL, creatine kinase 451U/L, CPK-MB fraction 20U/L, troponin <0.5 ng/mL, myoglobin 34.7 ng/mL, creatinine 1.2 mg/dL, urea 33 mg/dL, C-reactive protein 0.64 mg/dL, erythrocyte sedimentation time 38 seconds, serum aspartate tranaminase 187U/L and serum alanine transaminase 88.2U/L. Urine full report was normal. The transthoracic echo revealed a hypokinetic apex with an ejection fraction of 50% and evidence of dengue myocarditis. The serum IgM was positive for dengue and the serum IgG was negative.The following day, the pulse became regular and ECG showed sinus rhythm with no tachycardia (Figure 1B). The platelet count became normal and the patient had no arrhythmias on discharge on day 7.

The patient underwent a repeat ECG and Echocardiogram two months later. They were both normal. The Echo showed EF > 60% and there were no hypokinetic segments.

## Conclusion

Electrocardiographic abnormalities as a result of dengue infection are said to be in the range of 34–75% [4]. To the best of our knowledge, only one case of ventricular begeminy related to a possible dengue fever has been reported before [2]. In this case the clinical diagnosis of dengue fever was not made according to the WHO criteria for the diagnosis of dengue fever [5]. This makes our case the first ever reported case of ventricular trigeminy in a serologically confirmed case of dengue Infection. Ventricular trigeminy is a cardiac irregularity that consists of a continuous alternation of short and long cardiac cycles. Among the many causes for ventricular premature beats, this patient had no identifiable causes such as valvular heart disease, cardiomyopathy, electrolyte imbalances or acute myocardial infarction. He was not on any medications that could cause trigeminy. He denies consumption of cocaine, amphetamines, caffeine and alcohol. In our case, the ventricular trigeminy was considered to be due to dengue myocarditis as it occurred while the patient had the typical clinical picture of dengue fever consisting of thrombocytopenia, leukopenia, hemoconcentration and positive dengue serological tests. There was no previous history of palpitations or any symptoms of arrhythmia. His ECG and ECHO also did not show any evidence of structural heart abnormalities such as valvular heart disease, cardiomyopathy or ischaemic heart disease that may have predisposed him to develop ventricular arrhythmias.

The exact pathogenesis of dengue myocarditis is not known. It is postulated that alternation of autonomic tone and prolonged hypotension may play a role [6].

In conclusion, among many other known cardiac arrhythmia seen in DHF, ventricular trigeminy is also a possibility. Clinicians should be aware of this cardiac rhythm abnormality that can occur in dengue patients. Attention to these kinds of arrhythmias is necessary as it helps detect the underlying myocarditis which could lead to life threatening arrhythmias.

### Patients consent

The patient’s consent was obtained.

## Competing interests

The authors declare that they have no competing interests.

## Authors’ contributions

ATM, JI, SBG were involved in the management of the patient and drafting of the manuscript. All authors read and approved the final manuscript.
